# Interaction of light with gas–liquid interfaces: influence on photon absorption in continuous-flow photoreactors†

**DOI:** 10.1039/d4re00540f

**Published:** 2025-01-06

**Authors:** Jasper H. A. Schuurmans, Stefan D. A. Zondag, Arnab Chaudhuri, Miguel Claros, John van der Schaaf, Timothy Noël

**Affiliations:** a Flow Chemistry Group, van't Hoff Institute for Molecular Sciences (HIMS), Universiteit van Amsterdam (UvA) 1098 XH Amsterdam The Netherlands t.noel@uva.nl; b Department of Chemical Engineering and Chemistry, Sustainable Process Engineering, Eindhoven University of Technology (TU/e) 5612 AZ Eindhoven The Netherlands j.vanderschaaf@tue.nl

## Abstract

Light interacts with gas bubbles in various ways, potentially leading to photon losses in gas–liquid photochemical applications. Given that light is a valuable ‘reagent’, understanding these losses is crucial for optimizing reactor efficiency. In this study, we address the challenge of quantifying these interactions by implementing a method that separately determines the photon flux and utilizes actinometric experiments to determine the effective optical path length, a key descriptor of photon absorption. The results reveal the unexpected impact of gas phase introduction in continuous-flow photoreactors. Notably, photon absorption, and consequently the throughput of a photoreactor, can be increased by the introduction of a gas phase. This enhancement arises from the reflection and refraction effects of gas bubbles, which can intensify light intensity in the liquid volume and thereby offset any loss in residence time. The photon absorption losses that were observed were associated with large bubbles and were less significant than anticipated. In contrast, the introduction of small bubbles was found to increase photon absorption, suggesting it is a potential strategy to optimize photoreactor performance.

## Abstract 2

Light interacts with gas–liquid interfaces, yet photon absorption losses in a capillary and high-shear reactor are minimal. Reflection, refraction and scattering extend the optical path length, enhancing photon absorption when bubbles are small.

## Graphical abstract

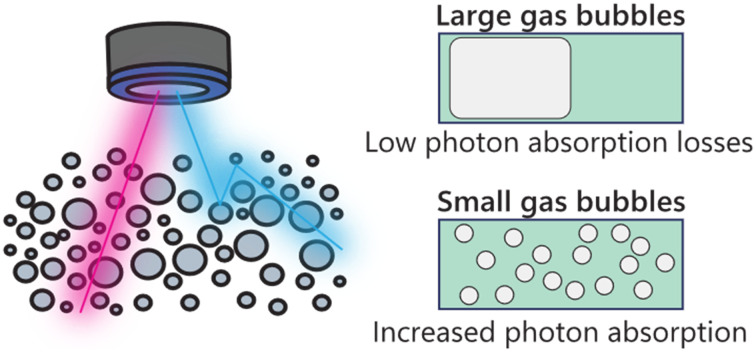

## Introduction

Continuous-flow reactors are widely employed for photochemical reactions due to their numerous advantages, including small dimensions and enhanced process control.^1–3^ The merging of a liquid reaction mixture with a gas phase in these reactors to form gas–liquid systems has significantly expanded the options for synthetic organic chemists, providing a means to mitigate gas–liquid mass transfer limitations.^4–6^ Gaseous reagents offer distinct benefits, being atom-efficient, cost-effective, and simplifying downstream separation processes. These advantages are demonstrated in various photochemical applications such as singlet oxygen chemistry, carbonylations, and light alkane activation.^7–14^ Additionally, inert gases are sometimes introduced to create segmented flow, improving mixing in the liquid phase. This approach has been shown to enhance residence time distribution properties and stabilize solid suspensions.^15–17^

In general, the use of gas–liquid systems in continuous-flow reactors has been shown to improve the performance of photochemical transformations.^17–21^ However, the incorporation of such multiphasic systems introduces additional complexity to photon absorption, as photons interact with gas–liquid interfaces.^22,23^ Small gas bubbles present in low volumetric quantities have been observed to have a minimal effect on photon absorption.^24,25^ Nonetheless, various characterization methods have reported different phenomena in intensified reactors, leading to a lack of consensus on the overall impact.^26–28^

The amount of photons absorbed in a photochemical system is a critical metric for assessing quantum yield, scaling up reactions, and designing energy-efficient reactors.^29–32^ However, accurately determining photon absorption in intensified gas–liquid systems presents a challenge due to the complexity of quantifying the fraction of unabsorbed, transmitted photons, which is necessary to in turn calculate the absorbed fraction from the incident photons. This challenge could be addressed by employing the radiative transfer equation or ray-tracing simulations tailored to the system's specific geometry.^33–38^ Yet, these methods require precise data on bubble size and position, which are not always readily available without extensive dedicated studies. A promising alternative to this complex geometrical determination is the use of the effective optical path length as a one-dimensional parameter to represent complex light interactions.^39^ This approach enables reliable assessment of transmitted photons in intensified gas–liquid systems and facilitates the characterization of various photoreactors under different hydrodynamic conditions.

**Fig. 1:**
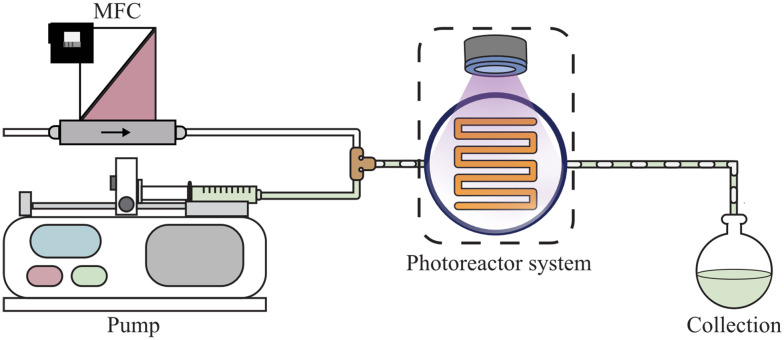
Schematic representation of the experimental setup for a gas–liquid continuous-flow photoreactor system, comprising a pump, mass flow controller (MFC), photoreactor system (including the reactor and light source), and a collection vessel.

For any photoreactor system, illustrated in Fig. 1, the effective optical path length can be determined by solving the mole balance. By selecting an appropriate actinometer, the corresponding balance can be constructed, with an ideal plug flow reactor represented by eqn (1).^40,41^ The key parameters in this balance include the concentration of the actinometer (*C*), the liquid flow rate (*Q*_l_, controlled by the pump), the photon flux (*q*, determined by radiometry and ray tracing^39^), the quantum yield (*φ*, specific to the reaction system), the Napierian absorption coefficient (*κ*, measurable by UV-VIS spectroscopy) and the effective optical path length (*l̄*, derived from actinometric experiments). In constructing the balance, gas bubbles are assumed to be non-absorbing and non-stagnant, resulting in the absence of the gas flow rate (*Q*_g_) and liquid residence time (*τ*_l_).^30,42,43^ Instead, the ratio of photon flux to inlet flow rate is used rather than liquid residence time. These assumptions imply that the photon flux incident on the reactor remains constant, with variations in photon absorption attributed solely to changes in the effective optical path length. In this work, the impact of the gas phase on photon absorption in gas–liquid photochemical transformations is investigated using both a microcapillary reactor and a high-shear reactor.^44,45^1
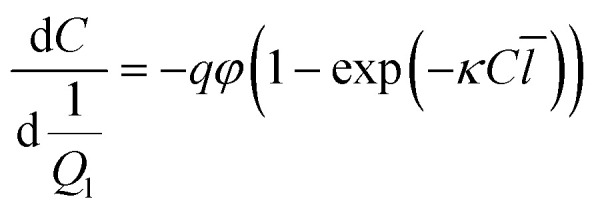


## Results

**Fig. 2:**
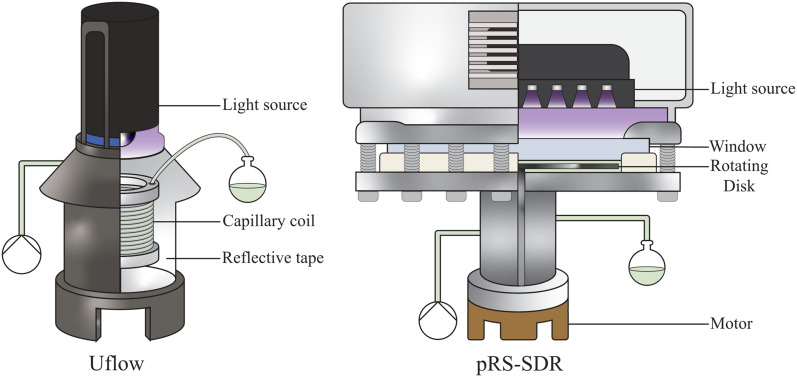
Schematic representation and cross section of the used microcapillary reactor (Uflow, 4.1 mL) and high shear reactor (photo rotor–stator spinning disk reactor (pRS-SDR), total volume 59 mL).

The potassium ferrioxalate actinometer (eqn (2)), a well-established standard, was employed to characterize the setups using ultraviolet UV-A (∼315–400 nm) light-emitting diode (LED) light sources.^46–49^ The actinometer conversion was measured at various flow rates to generate kinetic curves under each set of conditions. Nitrogen was introduced as an inert gas to examine the effect of gas phase addition on photon absorption, while gas-phase effects from carbon dioxide formation were neglected (Fig. S1b†). By utilizing photon flux data obtained *via* ray-tracing and radiometry, the effective optical path length was determined by fitting the kinetic curves to an appropriate reactor model.2
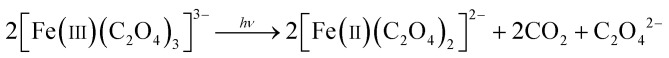
The microcapillary reactor used in this study is the open-source, 3D-printed Uflow reactor, as illustrated in Fig. 2.^45^ This photoreactor system consists of a capillary coiled around a holder, along with a light source and fan, all housed within a casing. The light source irradiates a cone, reflecting the light towards the capillary reactor. The holder and the interior of the casing are lined with reflective aluminum tape to maximize the incident photon flux on the reactor. The high-shear photoreactor used is the photo rotor–stator spinning disk reactor (pRS-SDR, shown in Fig. 2), which features a rotating disk enclosed in a closely fitting housing.^44,50^ The top section of the reactor is a quartz window, allowing for irradiation *via* an LED-based floodlight mounted in a stainless steel casing.^51^

## Photon absorption in a gas–liquid Uflow photoreactor

**Fig. 3:**
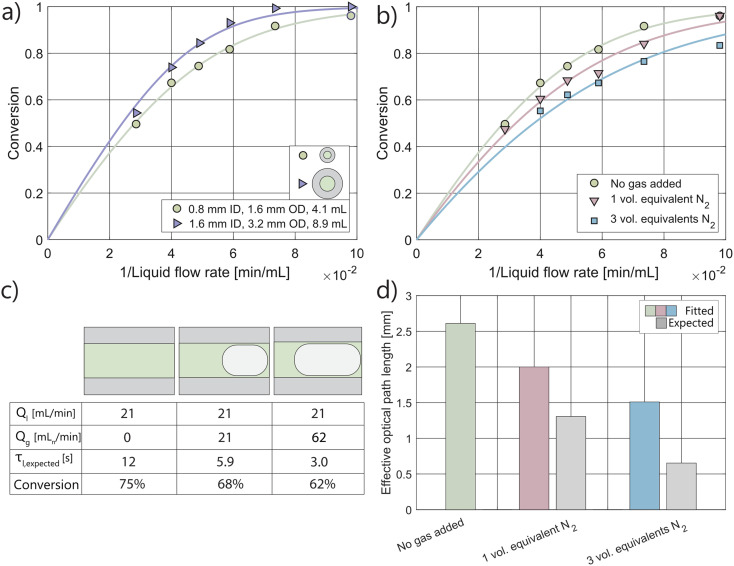
a) The kinetic curves and corresponding fits for the Uflow reactor (without any gas addition), using reactor coils with varying capillary size (ID inner diameter, OD outer diameter). b) The kinetic curves and corresponding fits for the Uflow reactor (4.1 mL), at various amounts of gas equivalents fed into the system. c) Selected results at different conditions, with a schematic of the expected volume contributions of the gas and liquid in the microcapillary (1/*Φ*_l_ of 4.9 × 10^−2^ min mL^−1^). d) The effective optical path lengths obtained from the kinetic curves, against the expected values based on the feed ratio.

The photoreactor setup, featuring the widely used Kessil light source (PR160L, 370 nm),^52–54^ incorporates a capillary reactor (4.1 mL, 1.6 mm outer diameter (OD), 0.8 mm inner diameter (ID)) coiled to span the full height of the holder. Considering the effects of refraction and the curvature of the capillary surface, the reactor's effective collection area can be extended to cover the entire height of the capillary, simplifying the determination of photon flux.^45,51,55^ Ray-tracing simulations confirmed that the total photon flux on the capillary is 1.9 μmol s^−1^, a value validated experimentally (see ESI† section S3.4).^45,56^ Using this data, the effective optical path length of the system, in the absence of gas, was determined by fitting the kinetic curve shown in Fig. 3, yielding a value of 2.6 mm.

Interestingly, this fitted path length is significantly larger than the inner diameter of 0.8 mm, suggesting that the setup using a capillary coil contributes to an extension of the optical path length, allowing light to pass the reaction volume multiple times. A different capillary (8.9 mL reactor volume, 3.2 mm OD, 1.6 mm ID) was tested under the same conditions, and similarly resulted in a path length much greater than the inner diameter (3.9 mm). Notably, the change in reactor volume induced by the different capillaries had a marginal effect on conversion (Fig. 3a). This is due to a proportional change in photon flux per reactor volume and comparable path lengths (2.6 mm and 3.9 mm). The use of a smaller capillary size effectively concentrates light on the reaction volume, significantly increasing space–time yield—achieving higher conversions at the same liquid residence time (Fig. S9b†). This observation confirms that the number of absorbed photons per unit of liquid flow rate, rather than per unit of liquid residence time, is the determining metric.^30^

Introducing an equivalent volume of gas into the system led to the formation of slug flow, characterized by alternating gas and liquid slugs.^57,58^ In this flow regime, a thin film of liquid can separate the gas bubbles from the capillary wall.^59,60^ As illustrated in Fig. 3b, introducing one equivalent volume of gas resulted in a decrease in photon absorption, which is attributed to a reduction in the effective optical path length. However, in terms of the feed ratio and actual gas holdup, photons are concentrated on the liquid inside the reactor. This is supported by a comparison with the expected path length, which serves as a reference point. The expected path length accounts for the volume occupied by the gas in the reactor, reducing the system's ability to absorb photons.^61^ Assuming an equal volumetric ratio of gas to liquid in both the feed and the reactor suggests that the original path length of 2.6 mm is reduced proportionally (*i.e.*, to 1.3 mm).

However, under the condition that the reactor model remains constant, photon absorption losses are mitigated, as the gas–liquid interfaces tend to interact with the light. This leads to similar performance levels at the same liquid flow rate, despite a significant reduction in liquid residence time (Fig. 3c). As the volumetric gas flow rate increases to a 3 : 1 gas-to-liquid feed ratio, the loss in photon absorption becomes more pronounced. These results indicate that larger gas bubbles are associated with a further reduction in the effective optical path length, as the flow regime remains in slug flow. Nonetheless, when comparing the expected path length based on the feed ratio to the fitted path length, it is evident that the decrease in photon absorption is significantly less than anticipated (Fig. 3d).

## Photon absorption in a gas–liquid photo rotor–stator spinning disk reactor

The pRS-SDR has a total reactor volume of 59 mL, with an irradiated volume of approximately 28 mL, located primarily between the quartz window and the rotating disk, which are separated by a 2.0 mm axial gap. The remaining volume, mainly beneath the disk, is considered non-irradiated. When the feed enters at the bottom of the reactor, the flow moves outward through the non-irradiated region (centrifugal flow) and inward in the irradiated region (centripetal flow).^62^ The photon flux for this system has been previously reported as 12.5 μmol s^−1^, which allows for the determination of the effective optical path length using an appropriate reactor model (using 3 CSTRs in series rather than 1 CSTR; see ESI† section S1.3). The fit obtained with this model gives an optical path length of 4.3 mm.^39^

**Fig. 4:**
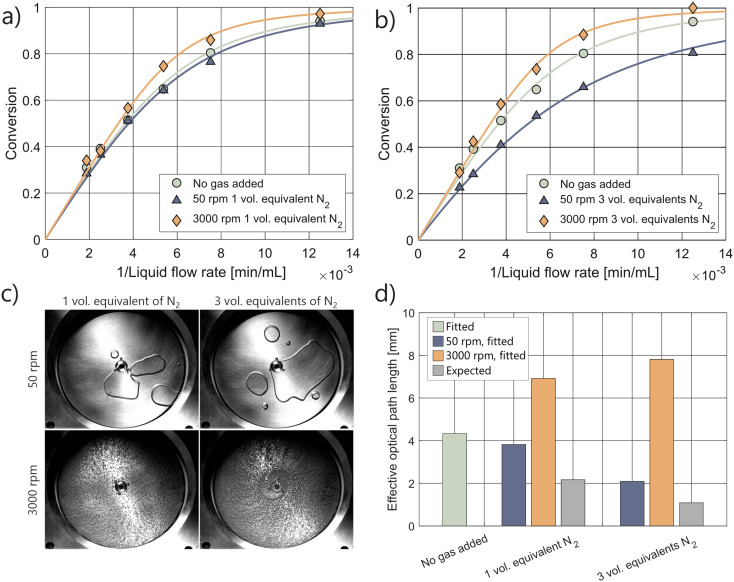
a and b) The kinetic curves and corresponding fits for the pRS-SDR reactor, at different rotation speeds and various amounts of gas equivalents fed into the system. Experiments without gas were conducted at a rotation speed of 1000 rpm. c) Representative images of the gas–liquid behavior in the irradiated part of the pRS-SDR at different rotation speeds and gas to liquid volumetric ratios. d) The effective optical path lengths obtained from the kinetic curves, against the expected values based on the feed ratio.

The behavior of gas bubbles in the pRS-SDR is highly dependent on the operating conditions. In the centrifugal flow region, a thin liquid film segregates a continuous gas phase from the liquid.^63–65^ In the centripetal flow region, a dispersed phase containing large bubbles (separated from the rotor and stator by a liquid film) and smaller bubbles is observed.^66^ The size of these bubbles in the dispersed region correlates with the rotation speed of the disk: higher rotation speeds lead to smaller bubbles, which can range within the micrometer scale.^44^Fig. 4c shows the formation of different bubble sizes when nitrogen is introduced into the actinometric system. At 50 rpm, large bubbles form, spanning most of the axial gap, while at 3000 rpm, smaller bubbles are generated due to the high shear forces exerted on the gas.^44,63^

The kinetic curves in Fig. 4 reveal that photon absorption is only slightly affected by the introduction of one equivalent of gas. At high rotation speeds (3000 rpm), photon absorption actually increases. The shortening of liquid residence time is almost fully counteracted, and in some cases even overcompensated, by the concentration of light on the liquid within the reactor. Similar to the Uflow reactor, the expected path length based on the feed ratio is significantly lower than the fitted path lengths (Fig. 4d). The lengthening of the effective optical path length at 3000 rpm is maintained even when the gas flow rate is increased to three times that of the liquid flow rate. At high rotation speeds, the addition of gas enhances photon absorption, thereby increasing the system's throughput.

At 50 rpm, a reduction in photon absorption and effective optical path length is observed, similar to the Uflow reactor, where large gas pockets span a large fraction of the axial gap. Gas holdup experiments indicate that the overall gas holdup increases with higher gas flow rates, and image analysis further confirms an increase in gas holdup within the irradiated region (Fig. S21†). These findings suggest that the decrease in photon absorption at lower rotation speeds can be correlated with the amount of gas in the bubbles in the upper part of the reactor.

## Discussion

Distinct cases with varying gas–liquid hydrodynamic behaviors were analyzed, revealing that photon absorption is highly dependent on the specific conditions employed. Gas bubbles create gas–liquid interfaces that interact with photons, but their influence on photon absorption in the liquid was found to be less significant than initially anticipated based on the feed ratio (Fig. 3d and 4d). Factors such as relatively low gas holdups, as well as reflection, refraction, and scattering, contribute to reducing photon absorption losses.^66–72^ The decrease in liquid residence time caused by the addition of a gas phase is counteracted by these effects, meaning that adding a gas phase does not require a proportional reduction in the liquid flow rate to maintain photon absorption at the same level.

Although the photon absorption losses were lower than expected, they were most pronounced in cases where gas bubbles nearly spanned the full axial length of the reactor. The results imply that a fraction of the photons which are initially transmitted after interacting with large gas bubbles find their way back into the reaction mixture. Total internal reflection can occur at the interface between the gas bubble and the reaction mixture, as well as between the reactor material (PFA or quartz) and the surrounding air. This allows the reactor material to function as a waveguide, preventing photon losses and enabling those photons to be absorbed upon re-entering the reaction mixture. The transition from large bubbles to smaller, spherical gas bubbles, as demonstrated in this study, led to increased photon absorption. Light rays scatter and change direction at these complex interfaces, so the introduction of smaller bubbles can further extend the effective optical path length.^73–76^

The results obtained apply to the specific experimental conditions and setup used, which include factors such as the light source, solvent, choice of (inert) gas, reactor material, and setup configuration. Nevertheless, these findings are likely to be relevant to other photoreactor systems and chemistries involving non-absorbing gases, similar reactor designs, bubble behavior, and comparable light source characteristics.

During the experiments, mass transfer effects were ruled out based on the absence of dark zones in the photoreactors, along with the use of high flow rates and/or rotation speeds (see ESI† section S1.4).^77–79^ Plug flow behavior was assumed for the microcapillary reactor, while a continuously stirred tank reactor (CSTR) model in series was applied to the high-shear reactor. These models were assumed to be valid across all operational conditions.^80^ Any variation in photon absorption due to changes in the reactor model is captured in the effective optical path length, which serves as an effective descriptor for photon absorption. This path length was obtained using photon fluxes determined through a combination of radiometry and ray-tracing.^39^

The methodology employed allows for the quantification of the average fraction of transmitted photons within the photoreactor system. The applicability of this method extends beyond the use of UV-A light and the potassium ferrioxalate actinometer. In principle, any actinometer and non-absorbing gas can be used, allowing for precise determination of the effective optical path length in most photoreactor systems. As demonstrated by the unexpected findings in this study, this characterization is crucial for accurately assessing performance and operational expenditures.^81–83^

## Conclusion

In conclusion, this study highlights the complex interplay between gas–liquid hydrodynamics and photon absorption in photoreactor systems. While gas bubbles introduce interfaces that can alter photon behavior, the anticipated photon absorption losses were less significant than expected, presumably due to mechanisms such as reflection, refraction, and total internal reflection. The results demonstrate that the introduction of a gas phase does not necessitate a proportional reduction in liquid flow rate to maintain efficient photon absorption. Smaller gas bubbles enhanced photon absorption by extending the effective optical path length through scattering and reflection effects. These findings, though specific to the experimental setup, have broader implications for optimizing gas–liquid photochemical processes, especially in systems involving non-absorbing gases. The methods used in this study, including the determination of the effective optical path length, offer a robust approach for evaluating and enhancing photoreactor performance across various configurations and conditions.

## Methods

### Chemical actinometry

The experimental procedure for potassium ferrioxalate actinometry was based on literature.^39,45–47^ A solution of 6.0 mM of potassium ferrioxalate (Alfa Aesar/Thermo Scientific) in 50 mM sulfuric acid (Sigma Aldrich) solution, using demineralized water as solvent, was prepared. The solution was pumped through the Uflow and pRS-SDR at selected liquid flow rates. Gases were supplied with a mass flow controller (MFC, Bronkhorst) and the gas and liquid phase were mixed using a T-mixer. Gas equivalents are based on the volume under normal conditions (0 °C and 1 atm). The mixture was irradiated with a UV-light source (Uflow: Kessil PR160L 370 nm, input power 43 W, operated at 25% power intensity, pRS-SDR: UV-A curing 365 nm floodlight, input power 175 W). Sampling at the outlet was done at steady-state conditions (*i.e.*, after waiting at least two residence times). The microcapillary reactor (PFA, 1.6 mm outer diameter, 0.8 mm inner diameter, 4.1 mL) was not operated in stop-flow to increase the mixing and prevent unwanted dilution.^77,78^ Blank samples were taken after each experiment to account for any conversion of the actinometer caused by background irradiation.

The samples (0.1 mL) were diluted with water (1.4 mL) and after dilution 0.6 mL was added to a buffer solution (2.0 mL). The buffer consisted of 6 mM 1,10-phenanthroline (Sigma Aldrich), 0.6 M sodium acetate (Sigma Aldrich) in 0.18 M sulfuric acid, using demineralized water as solvent, consistent to the procedure reported.^39,45^ The samples were measured using a UV-VIS spectrophotometer (UV-2501 PC Shimadzu or Horiba Scientific Duetta) in a 1 cm cuvette and the peak absorption of the complex at 510 nm was noted and used to determine the conversion, using calibration curves.

### Radiometry

Radiometric characterization of the light sources was previously conducted and the resulting data was applied in the current study.^39^

### Ray-tracing

To develop the light source and reactor setup simulations, geometric representations of the light sources and reactors were constructed based on the measured dimensions, as detailed in prior research.^39,45^ These representations, along with the material properties were then modeled in COMSOL Multiphysics 5.4. Calibrated models of the light sources, and the model of the pRS-SDR and Uflow were obtained from previous work and simulated using the Geometric Optics module.^39,45^

### Non-linear fitting of the effective optical path length

The mole balance for each reactor system was implemented in MATLAB R2022b. A non-linear least squares method was used to fit the effective optical path length in the balance. The differential equations were solved with a variable-step, variable-order (VSVO) solver for stiff differential equations.

### Workflow

The applied workflow to obtain the photon flux and effective optical path length is given by prior research.^39^

### Imaging of the pRS-SDR

Images of the pRS-SDR supplied in the main text (Fig. 4c) were recorded with a high-speed camera (SpeedSense, Dantec Dynamics). A sample rate of 200 pulses per second was used, with exposure times ranging from 150 μs to 1000 μs and an extended dynamic range (EDR) from 100 μs to 500 μs, based on the employed rotation speed. A solution consisting of 6.0 mM of potassium ferrioxalate in 50 mM sulfuric acid was used for the imaging to ensure conditions equal to the operating conditions. Representative images were selected for each condition.

## Data availability

The data supporting this article have been included as part of the ESI.†

## Conflicts of interest

There are no conflicts to declare.

## Supplementary Material

RE-010-D4RE00540F-s001
