# Supplementary material for: Interaction of light with gas–liquid interfaces: influence on photon absorption in continuous-flow photoreactors
Source: React Chem Eng. 2025 Jan 6;10(4):790–9. doi: 10.1039/d4re00540f (PMC11726180; doi:10.1039/d4re00540f)
Supplement: RE-010-D4RE00540F-s001 [file RE-010-D4RE00540F-s001.pdf]

Supplementary Information (SI) for Reaction Chemistry & Engineering.
This journal is © The Royal Society of Chemistry 2025

Interaction of Light with Gas-Liquid Interfaces: Influence on Photon Absorption in Continuous-Flow Photoreactors

Supplementary Information

Jasper H.A. Schuurmans,[1] Stefan D.A. Zondag,[1] Arnab Chaudhuri,[2] Miguel Claros,[1] John van der Schaaf,[2],* Timothy Noël[1],*

[1] Flow Chemistry Group, van't Hoff Institute for Molecular Sciences (HIMS), Universiteit van Amsterdam (UvA), 1098 XH Amsterdam, The Netherlands.

[2] Department of Chemical Engineering and Chemistry, Sustainable Process Engineering, Eindhoven University of Technology (TU/e), 5612 AZ Eindhoven, The Netherlands.

* Corresponding Author(s): t.noel@uva.nl (Timothy Noël) and j.vanderschaaf@tue.nl (John van der Schaaf)

Contents

S1. Effective optical path length determination 3

S1.1 General 3

S1.2 Uflow 3

S1.3 pRS-SDR 3

S2. Imaging of the microcapillary reactor 5

S3. Additional information Uflow 7

S3.1 Reactor setup 7

S3.2 Gas holdups 7

S3.3 Capillary size 8

S3.4 Reflectivity reactor casing and photon flux validation 9

S4. Imaging of the pRS-SDR 12

S5. Additional information pRS-SDR 16

S5.1 Reactor setup 16

S5.2 Gas holdups 16

Symbol list 18

References 19

S1. Effective optical path length determination

S1.1 General

The method to determine the optical path length for a photochemical reactor system is based on previous work.[1] In constructing the balances, the wavelength dependency of the absorption coefficient is taken into account, while the quantum yield is assumed to remain constant across the wavelength range under the specified conditions.[2] Additionally, the spectral distribution of the specific light source was included in the balance. The final balance of a batch reactor system is given by Equation S1.[1] The absorption of the formed products is assumed to be negligible.[3]

$$\frac{dN_A}{dt} = - q_{n,p}\varphi \sum_{\lambda_1}^{\lambda_{end}} g_{p,\lambda}\left(1 - \exp\left(- \kappa_{A,\lambda}C_A\bar{l}\right)\right) \quad \text{S1}$$

Balances for continuous-flow systems can be constructed in a similar manner. The addition of a gas phase can add complexity when the balance needs to be solved with respect to the residence time. In this case, the exact liquid volume in the system needs to be acquired, or one can choose to use the photon flux per liquid volume. For the latter, the overall photon flux should remain equal for all conditions, given that bubbles are non-stagnant. To reduce complexity, the mole balance can be formulated in terms of concentration and liquid flow rate, completely omitting the (liquid) reactor volume from the balance. To use a balance and make a fit on a data set, it is necessary to assume that the effective optical path length is independent of the absolute flow rates within that specific set. The actual concentration at the outlet for each system is determined with the made calibration curves (ref. 1 and Figure S1a).

The release of carbon dioxide by the actinometric reaction is assumed to have a negligible effect on the overall gas holdup, based on experiments conducted in a batch reactor (1:1 gas to liquid ratio). Several initial concentrations of the actinometer were reacted until full conversion was obtained, after which the headspace was sampled (analyzed by gas chromatography, method based on ref. 4). The carbon dioxide tends to remain dissolved in the liquid shortly after and during the reaction, based on differing results at different time intervals (Figure S1b).

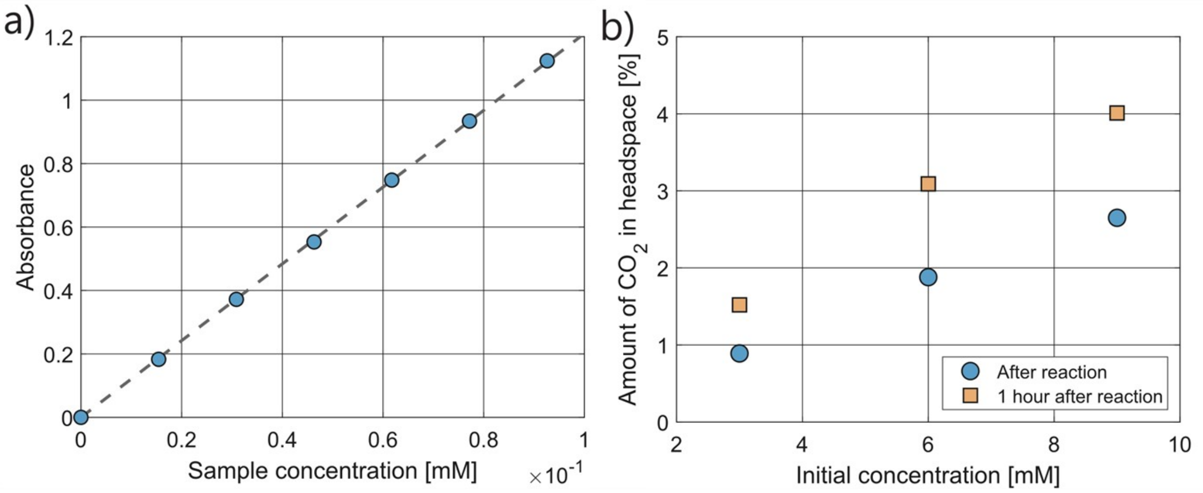

Figure S1 a) The calibration curve showing the absorbance of the phenanthroline Fe(II) complex, against the sample concentration (Slope equals 12.1 [$mM^{-1}cm^{-1}$]). b) The volume percentage of carbon dioxide in the headspace of a batch reactor after fully converting a varying concentration of actinometer.

S1.2 Uflow

The microcapillary reactor was assumed to behave as a plug flow reactor under all investigated conditions, giving rise to Equation S2.

$$\frac{dC_A}{d\frac{1}{Q_l}} = - q_{n,p}\varphi \sum_{\lambda_1}^{\lambda_{end}} g_{p,\lambda}\left(1 - \exp\left(- \kappa_{A,\lambda}C_A\bar{l}\right)\right) \quad \text{S2}$$

S1.3 pRS-SDR

Several studies have investigated the selection of the reactor model for the rotor-stator spinning disk reactor. The reactor is typically modelled as a combination of a plug flow reactor and continuously stirred tank reactor (CSTR), depending on the employed operating conditions.[5–7] Nevertheless, the addition of a gas phase and the division between an irradiated and non-irradiated part complicates the choice of the reactor model. Hereto, a CSTRs in series model is employed to describe any non-ideal behavior in the pRS-SDR.[8] The actual number of CSTRs in series (N) was determined based on residence time distribution experiments,[7] and the fit on the data obtained for the pRS-SDR. The residence time distribution data (Figure S2a) was obtained for the full reactor at 1002 rpm and a liquid flow rate of 591 mL/min.[7] The root mean square error (RMSE) for the different number of tanks in series shows a decrease until 4 tanks, whereafter it increases. The fit on the actinometric data obtained (1000 rpm, no gas) and the RMSE of the fit with a varying number of tanks in series (Figure S2c and d) show a similar trend, where the increase in error already occurs after 2 tanks in series. Based on the insights obtained, it was decided to use a model employing 3 CSTRs in series to fit the effective optical path length. The general balance is given by Equation S3, where i represents the inlet to tank $i + 1$. The reactor model assumes that the tanks all have the same volume, so the photon flux was divided by the total number of CSTRs. The balances were solved iteratively in MATLAB R2022b employing a combination of bisection, secant and inverse quadratic interpolation methods. The model is assumed to be valid over the full range of investigated conditions. Any changes in photon absorption caused by a change in reactor model at differing conditions are captured in the effective optical path length for both the Uflow and pRS-SDR.

$$\frac{C_{A,i} - C_{A,i+1}}{\frac{1}{Q_l}} = -\frac{q_{n,p}}{N}\varphi\sum_{\lambda_1}^{\lambda_{end}} g_{p,\lambda}\left(1 - \exp\left(-\kappa_{A,\lambda}C_{A,i+1}l\right)\right) \qquad \text{S3}$$

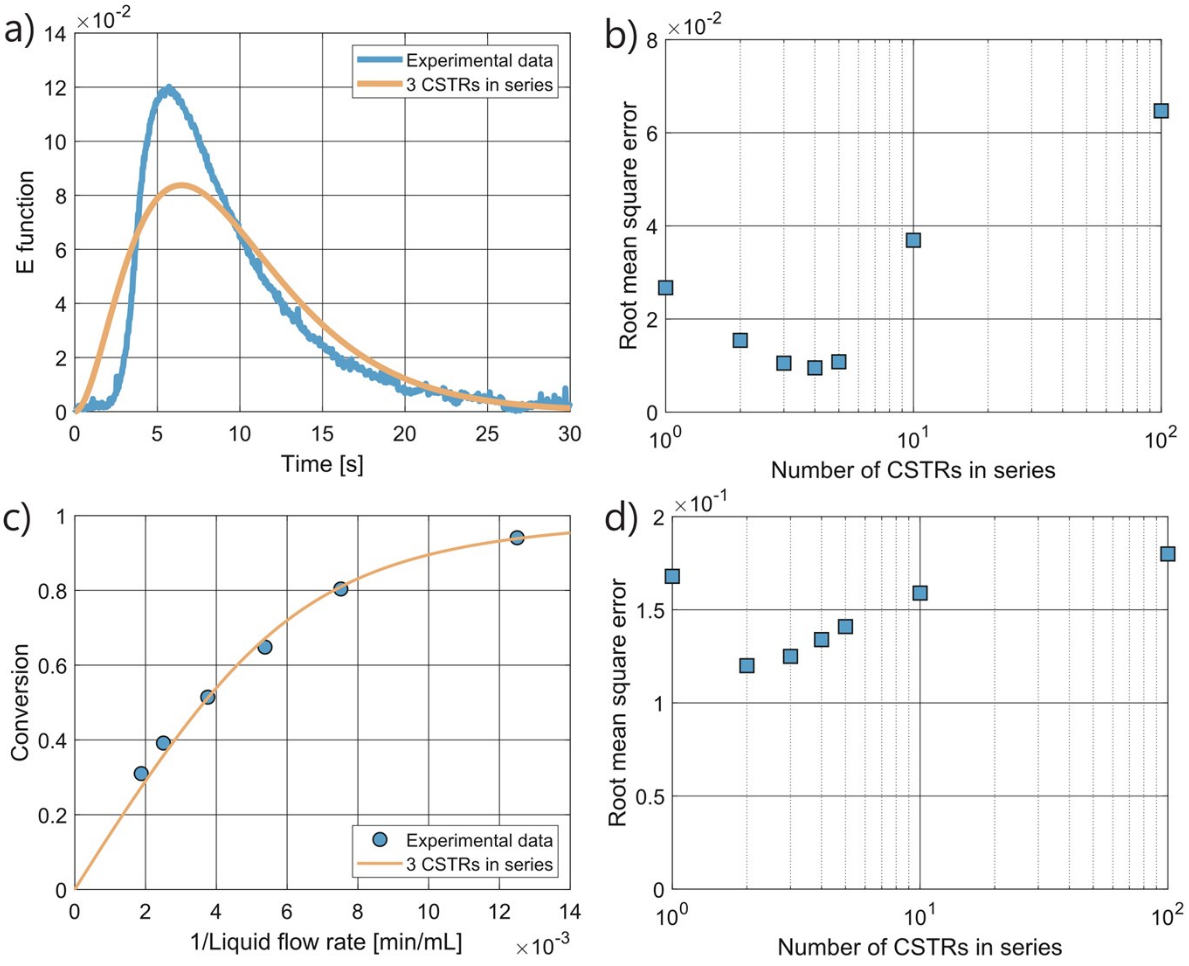

Figure S2 a) Experimental residence time distribution (RTD) data[7] for the full RS-SDR, showing the fit with the chosen reactor model. b) The root mean square error (RMSE) for different number of continuously stirred tank reactors (CSTRs) in series fitted on the RTD data. c) The kinetic curve and corresponding fit for the pRS-SDR reactor. d) The RMSE for different number of CSTRs in series fitted on the kinetic data.

S1.4 Mass transfer

When constructing the mole balances and interpreting the results any mass transfer effects were ruled out. For the chosen photoreactor systems any by-pass of the actinometer without interacting with light is not feasible. This suppresses potential limitations caused by mixing between permanent dark and irradiated zones.[9] In the irradiated zones, the only strongly absorbing species is the actinometer. The absence of competitive absorbing species further reduces the need for mixing.[10,11] Additionally, relatively high flow rates were employed, ensuring sufficient mixing in the system.[12] Previously conducted experiments indicated that for the pRS-SDR similar results were obtained for rotation speeds varying from 100 to 2000 rpm, further supporting the assumption that no mass transfer limitations occur.[1]

S2. Imaging of the microcapillary reactor

The imaging process to capture the gas-liquid behavior within the capillary reactor involved several steps. First, the appropriate gas and liquid flow rates were set, followed by the establishment of steady state by waiting several residence times. Hereafter, the reactor was closed using shut-off valves at the inlet and outlet. This approach was necessary due to the high velocities within the capillary, which made it difficult to obtain clear and sharp images. Nevertheless, this approach can lead to an increase in the coalescence of slugs, explaining the variability of the slug sizes in Figure S3 and Figure S4.

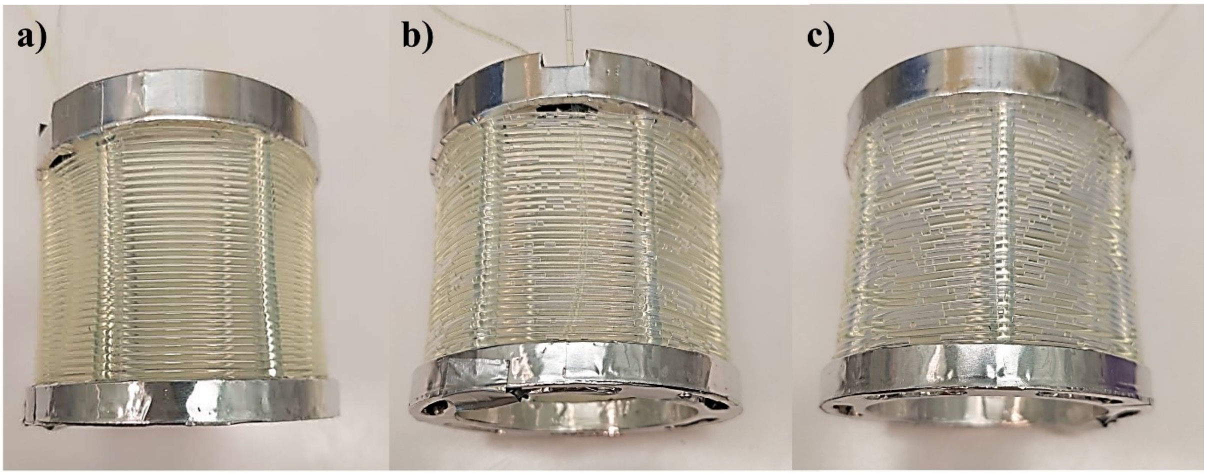

Figure S3 The used capillary reactor, with representative images for situations without gas (a), with 1 vol. equivalent of nitrogen (b), and with 3 vol. equivalents of nitrogen (c). Images were made with a higher actinometer concentration to increase the contrast.

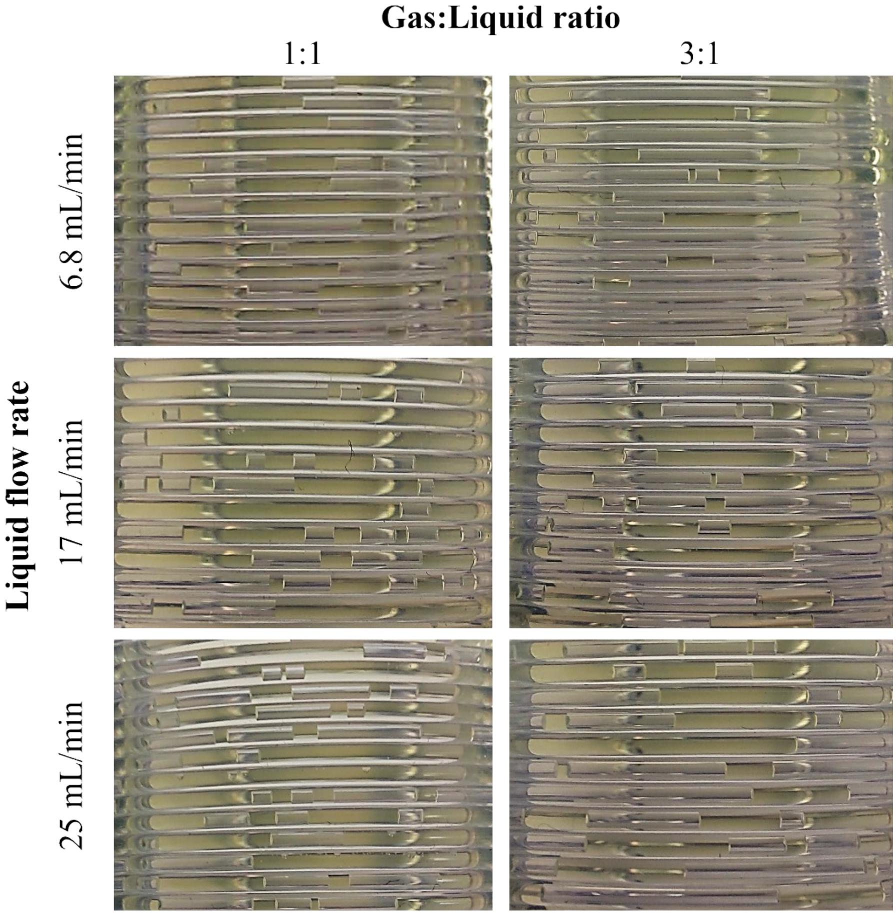

Figure S4 Example images of the gas-liquid behavior under various flow rates and gas-liquid ratios. Images were made with a higher actinometer concentration to increase the contrast.

S3. Additional information Uflow

S3.1 Reactor setup

Comprehensive details regarding the Uflow reactor are available in literature.[13] The Uflow reactor used in this research was printed with gray polylactic acid (PLA) and the inside was fully covered with aluminum tape. The setup for the reactions (Figure S5) consisted of a syringe pump (Chemyx) and mass flow controller (MFC, Bronkhorst). As detailed in previous research, the used light source (Kessil PR160L 370nm) was characterized.[1]

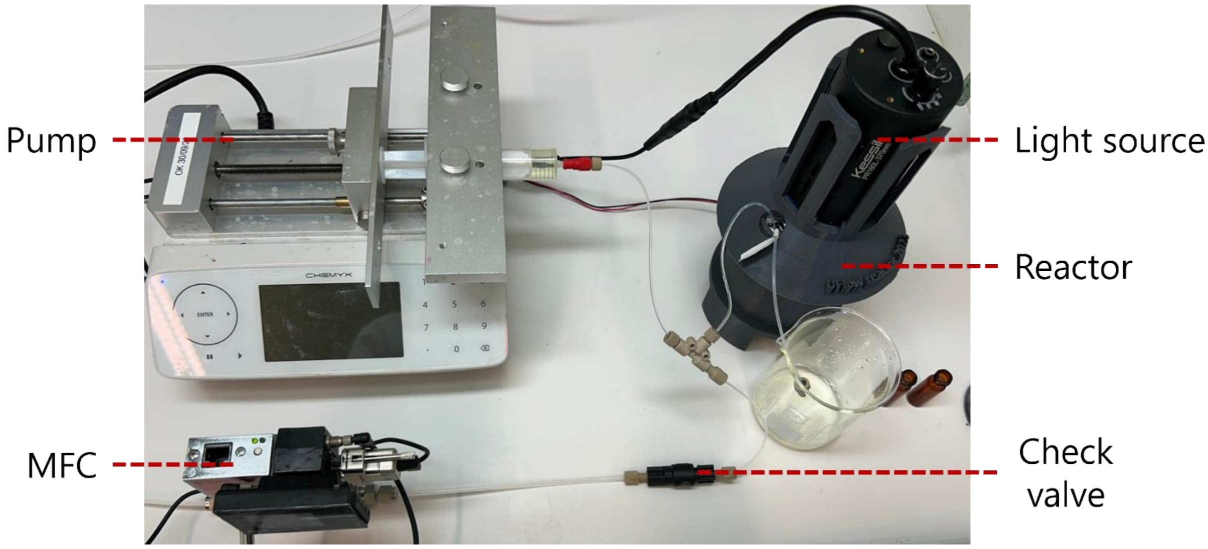

Figure S5 Overview of the setup used to perform actinometry experiments in the Uflow reactor.

S3.2 Gas holdups

To gain deeper insights into the gas-liquid behavior within the microcapillary reactor, gas holdups were measured under various conditions. The holdup was measured continuously using the setup illustrated in Figure S6. The coil was placed on a scale and completely filled with actinometric solution, after which the weight was noted. Hereafter, the desired flow rates on the pump and MFC were set. The weight difference registered can be correlated to the volume of liquid that is replaced with gas and can be used to calculate the gas holdup. The calculations assumed a negligible weight contribution of the gas. Actinometric solution was used to work as close as possible to operating conditions. Figure S7 shows that the gas holdup increases with higher amounts of nitrogen added, while it shows a downward trend for identical feed ratios with increasing liquid flow rate. This trend is dictated to the elevated pressure drop at higher flow rates, causing increased compression of the gas. Overall, within the standard operating regime (6.8 to 25 mL/min), similar gas-liquid behavior is observed when the gas-to-liquid volumetric ratio is maintained constant.

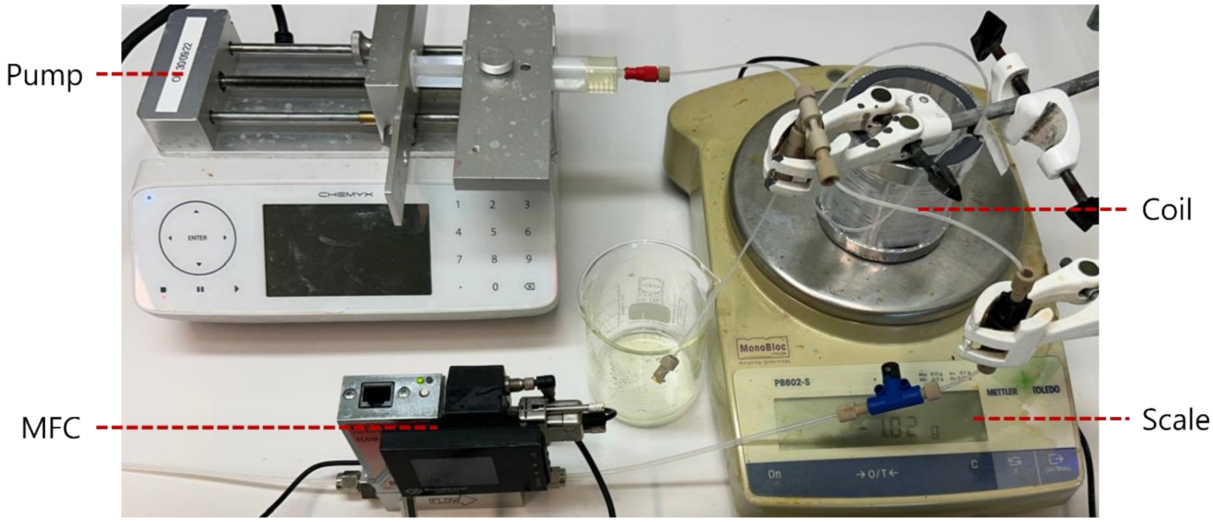

Figure S6 Overview of the setup used to perform the holdup experiments in the Uflow coil.

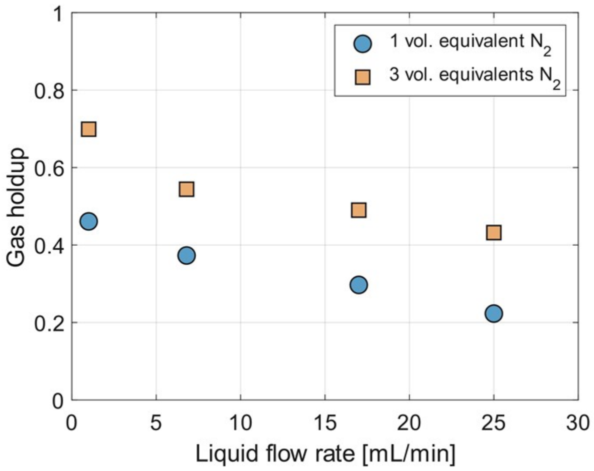

Figure S7 The average gas holdup of the coil used in the Uflow at different gas and liquid flow rates.

S3.3 Capillary size

As indicated in Figure S8, various capillary sizes on the same holder have been investigated. Based on the refraction and curvature of the PFA tubing, it was assumed that all photons incident on the tubing will interact with the reaction mixture, leading to this flux to be considered the maximum incident photon flux.[1,14] Figure S9a shows the kinetic curves for the different tube sizes. It can be noted that the changes in conversion are not proportional to the large change in volume between the reactors. It appears that photons are indeed concentrated on the reaction volume, where a decrease in volume leads to an increase in photon flux per reactor volume. The only descriptor of the conversion in this case is the effective optical path length of the individual systems, which were found to be 1.1, 2.6 and 3.9 mm, for the three configurations in Figure S8, respectively. These results indicate that there is a balance between the incident photons per reactor volume and the absorbed fraction of these photons (determined by the effective optical path length). Overall, the space-time yield can increase vastly by using smaller capillary sizes, which effectively concentrates photons on the reaction mixture (Figure S9b). Nonetheless, small inner diameters in combination with high flow rates can lead to an excessive pressure drop, posing an additional design consideration for the construction of a microcapillary reactor.

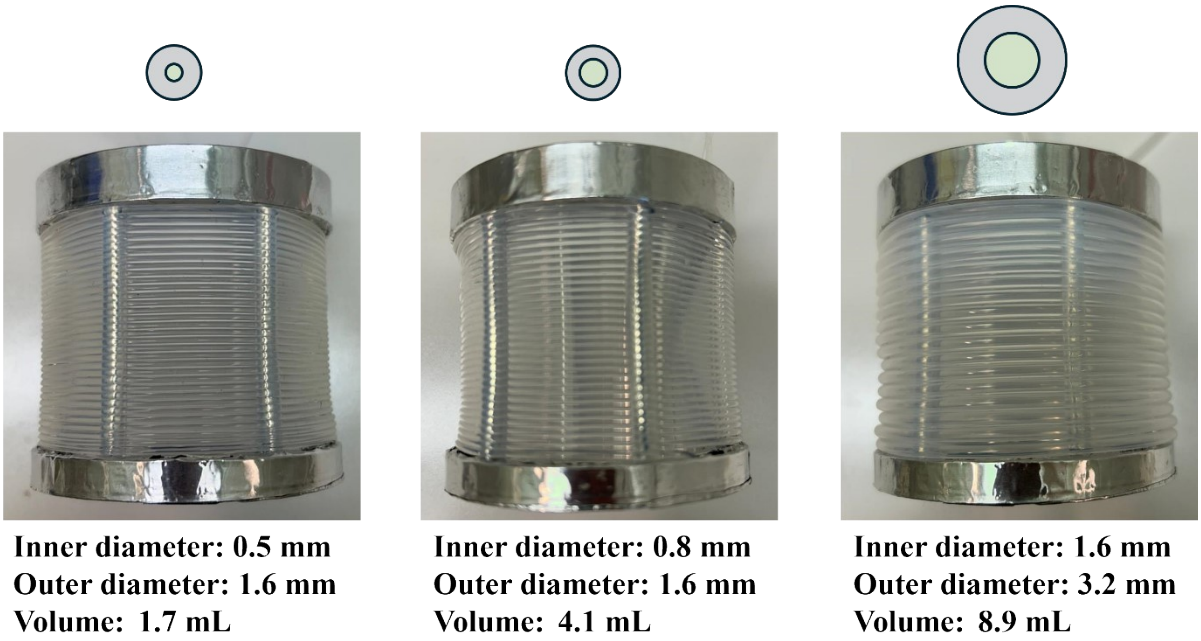

Figure S8 The three different capillary sizes used for the actinometric experiments in the Uflow reactor.

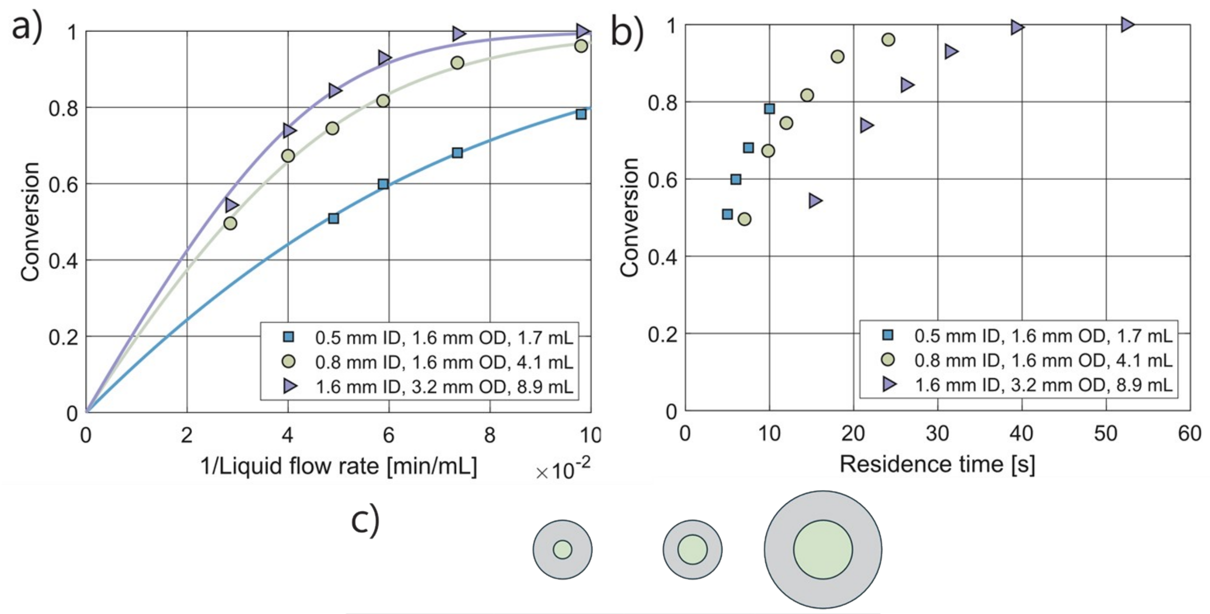

Effective optical path length	1.1 mm	2.6 mm	3.9 mm
Path length to ID ratio	2.2	3.3	2.4
Initial reaction rate	1.33 $\frac{mol}{s}$	2.05 $\frac{mol}{s}$	2.23 $\frac{mol}{s}$
Initial reaction rate per volume	0.78 $\frac{mol}{m^3 \cdot s}$	0.50 $\frac{mol}{m^3 \cdot s}$	0.25 $\frac{mol}{m^3 \cdot s}$

Figure S9 a) The kinetic curves and corresponding fits for the Uflow reactor, using reactor coils with varying capillary sizes (ID, inner diameter, OD outer diameter). b) The kinetic curves against the liquid residence time. c) The results obtained from the fits for the different capillary sizes.

S3.4 Reflectivity reactor casing and photon flux validation

Experiments in a batch reactor type (UFO[13] reactor, with a Kessil light source; PR160L 370nm, at 25%), with glass tubes (Pyrex, inner diameter 13.2 mm, outer diameter 15.2 mm, filled with 2 mL solution) were used to investigate the effect of reflective tape on the PLA reactor casing. These experiments pointed out that the use of the reflective tape can increase the received photon flux significantly (see Figure S10). The incident photon flux in each scenario can be fitted for data points with low conversion, assuming full photon absorption, which for the batch glass tubes is ~60% conversion before this assumption is invalid.[1] Deriving the mole balance for the batch reactor results in a version of Equation S4 dependent on reaction time and volume. The batch experiments were performed in duplicate, using a setup with 4 glass tubes filled with 2 mL of actinometric solution each. The resulting photon flux per tube for the situation with reflective tape (0.171 µmol/s) was found to be three times higher than in the scenario without it (0.059 µmol/s). The system using reflective tape was simulated using COMSOL Multiphysics 5.4's Geometric Optics module for the glass tubes filled with 2 mL, using a reflective loss of 7.5%, as previously reported in ref. [13] for the tubes filled with 4 mL, assuming specular reflection. The photon flux determined using the simulation was found to be 0.168 µmol/s (vs. 0.171 µmol/s experimentally). This good agreement allowed for an estimate of the reflective loss of the gray PLA using the experimental flux for the bare PLA casing. Assuming either absorption or diffuse reflection upon interacting with the material ~76% of optic power is lost to the PLA, allowing ~24% to reflect. In previous work this reflectivity was assumed to be 0, which for this system would have resulted in an expected photon flux of 0.039 µmol/s, significantly lower than the experimental result of 0.059 µmol/s.

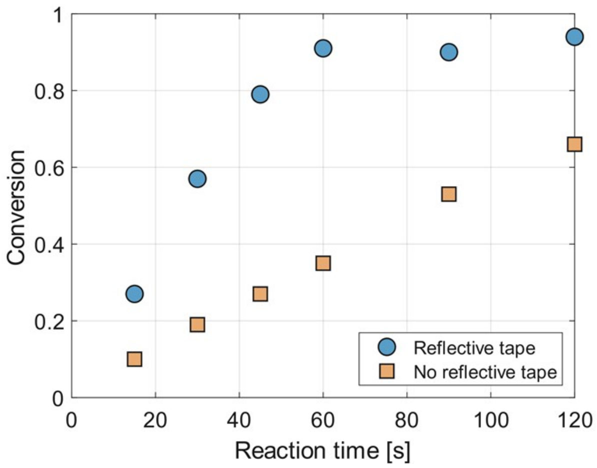

Figure S10 The conversion against the reaction time in two distinct UFO reactors, one with reflective tape and one without reflective tape, using 4 glass tubes filled with 2 mL.

This found reflectivity was then applied to the simulations of the Uflow reactor. In the same manner as for the batch experiments and simulations, two versions of the reactor system have been made: one reflective, and one with bare PLA. For the reflective system, the inside of the gray PLA casing was fully covered with reflective tape, as was the capillary holder. In the previously reported work,[13] the inside of the casing was only partly covered with tape, and the capillary holder was bare PLA. This notable difference is expected to increase the photon flux received by the capillary. For the reflective system (assuming again specular reflection with a 7.5% reflective loss) the resulting photon flux was 1.95 µmol/s, almost twice as high as for the bare PLA, which resulted in 1.02 µmol/s (assuming diffuse reflectivity and a 76% reflective loss).

The photon flux determined through radiometry and ray-tracing simulations assumes that all photons incident on the capillary end up interacting with the reaction mixture.[1,13] The assumption can be validated by performing the chemical actinometry experiments, reported in the Method section, with higher concentrations of the actinometer. An initial concentration of 150 mM was employed to allow for the assumption of complete photon absorption at low conversions, leading to Equation S4 (initially $\kappa_A C_A \approx 2 \cdot 10^4$ m^{-1} describing a photon-efficient system for path lengths > 0.2 mm).[1,15] Fitting the experimental data (Figure S11) results in a experimentally determined photon flux of 1.9 µmol/s, which shows excellent agreement with the number obtained with radiometry and ray-tracing simulations. The quantum yield was assumed to be unaffected by the increase in concentration.[16]

$$\frac{\Delta C_A}{\Delta \frac{1}{Q_l}} = - q_{n,p} \varphi \qquad \text{S4}$$

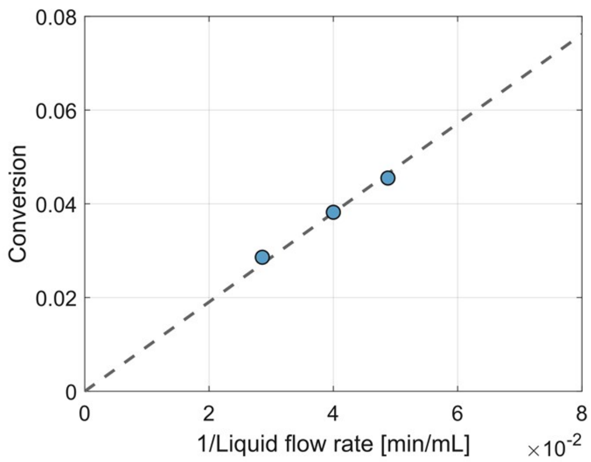

Figure S11 The kinetic curve and corresponding fit for the Uflow reactor (4.1 mL, ID 0.8 mm, OD 1.6 mm) with a starting concentration of 150 mM.

With the photon flux assumptions validated, the difference between the reflective and bare PLA Uflow reactors, together with the experimental data (see Figure S12), allowed for the fitting of the effective optical path lengths for both systems. As can be seen in Table S1 (using fluxes of 1.95 and 1.02 µmol/s for reflective tape and bare PLA systems, respectively), there only is a minor difference in fitted effective optical path lengths, and all are significantly larger than expected based on their inner diameter of 0.8 mm. The fluxes vary by almost a factor of two, and the additional diffuse or specular reflections do affect the direction of light incident on the capillary. This implies that the effective optical path length in this reactor system is governed by the capillary coil's size and shape, and less by the reactor casing, that only affects the magnitude of the received flux.

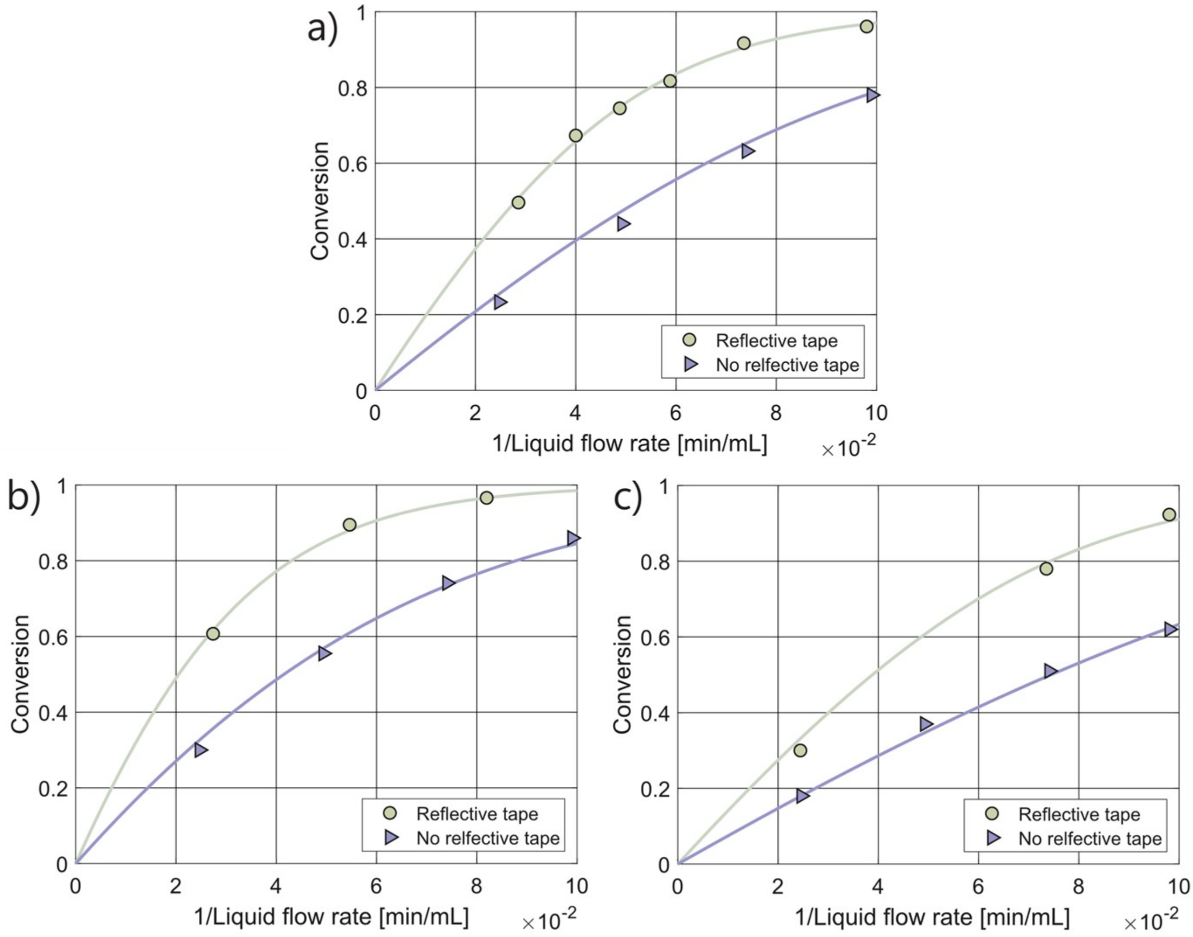

Figure S12 The kinetic curves and corresponding fits in two distinct Uflow reactors (both 4.1 mL, ID 0.8 mm, OD 1.6 mm), one with reflective tape and one without reflective tape, employing various initial actinometer concentrations (a) 6.0 mM, b) 3.0 mM and c) 9.0 mM).

Table S1 The fitted effective optical path length for distinct starting concentrations in the Uflow (4.1 mL, ID 0.8 mm, OD 1.6 mm), utilizing the corresponding photon fluxes for the situations with and without reflective tape in the casing.

Initial actinometer concentration [mM]	*Reflective tape in casing*	*Effective optical path length [mm]*
6.0	*Yes*	*2.6*
6.0	*No*	*2.7*
3.0	*Yes*	*2.7*
3.0	*No*	*2.5*
9.0	*Yes*	*2.1*
9.0	*No*	*2.0*

S4. Imaging of the pRS-SDR

The image analysis of the pRS-SDR was performed with the setup shown in Figure S13, actinometric solution and nitrogen were used, with several images recorded to obtain representative images under each condition. As detailed in the method section, a high speed camera (SpeedSense, Dantec Dynamics) was used. The images (Figure S14 to Figure S19) indicate that the bubble size in the top part of the reactor correlates strongly with the rotation speed. Moreover, the gas-liquid behavior appears to be relatively independent of the absolute liquid flow rate employed, for all rotation speeds investigated. Increasing the volume equivalents of nitrogen gives visually rise to an increased number of larger bubbles in the top part of the reactor for the investigated rotation speeds below 3000 rpm.

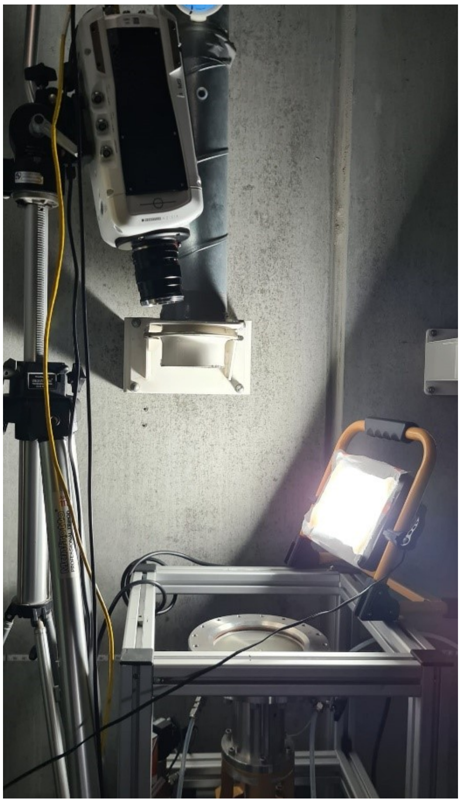

Figure S13 Setup used for the imaging of the gas-liquid behavior in the pRS-SDR.

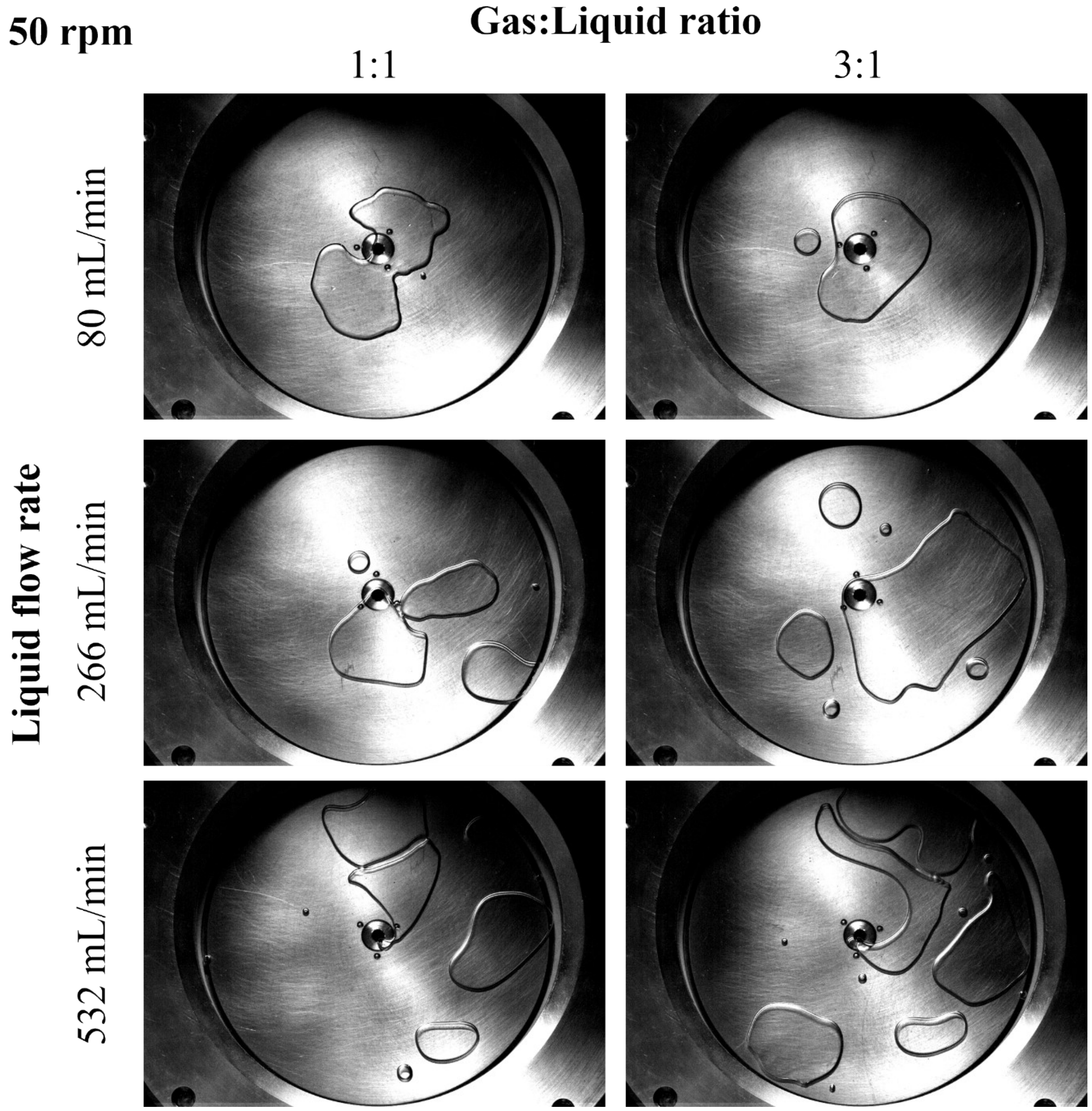

Figure S14 Representative images of the gas-liquid behavior under various flow rates and gas-liquid ratios, at a rotation speed of 50 rpm.

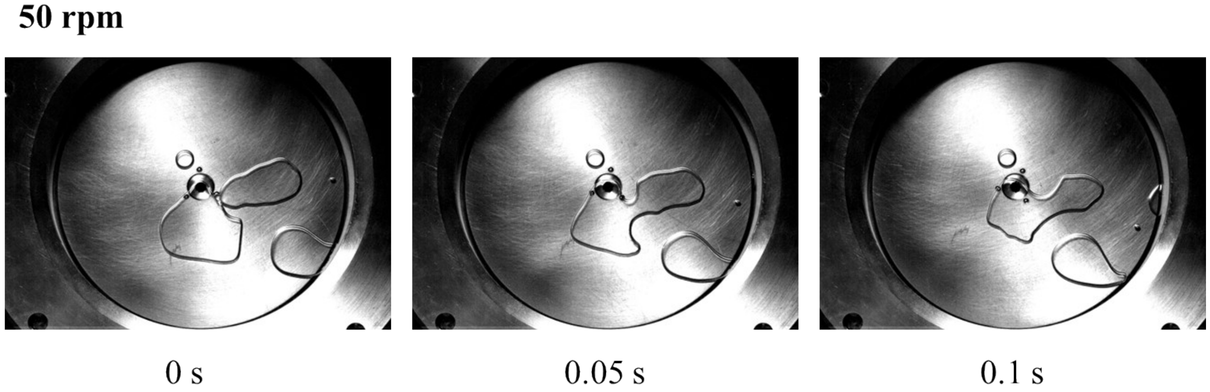

Figure S15 Timelapse of the irradiated part of the pRS-SDR at a rotation speed of 50 rpm, liquid flow rate of 266 mL/min and 1 vol. equivalent of nitrogen, showing 0.08 rotation of the disk.

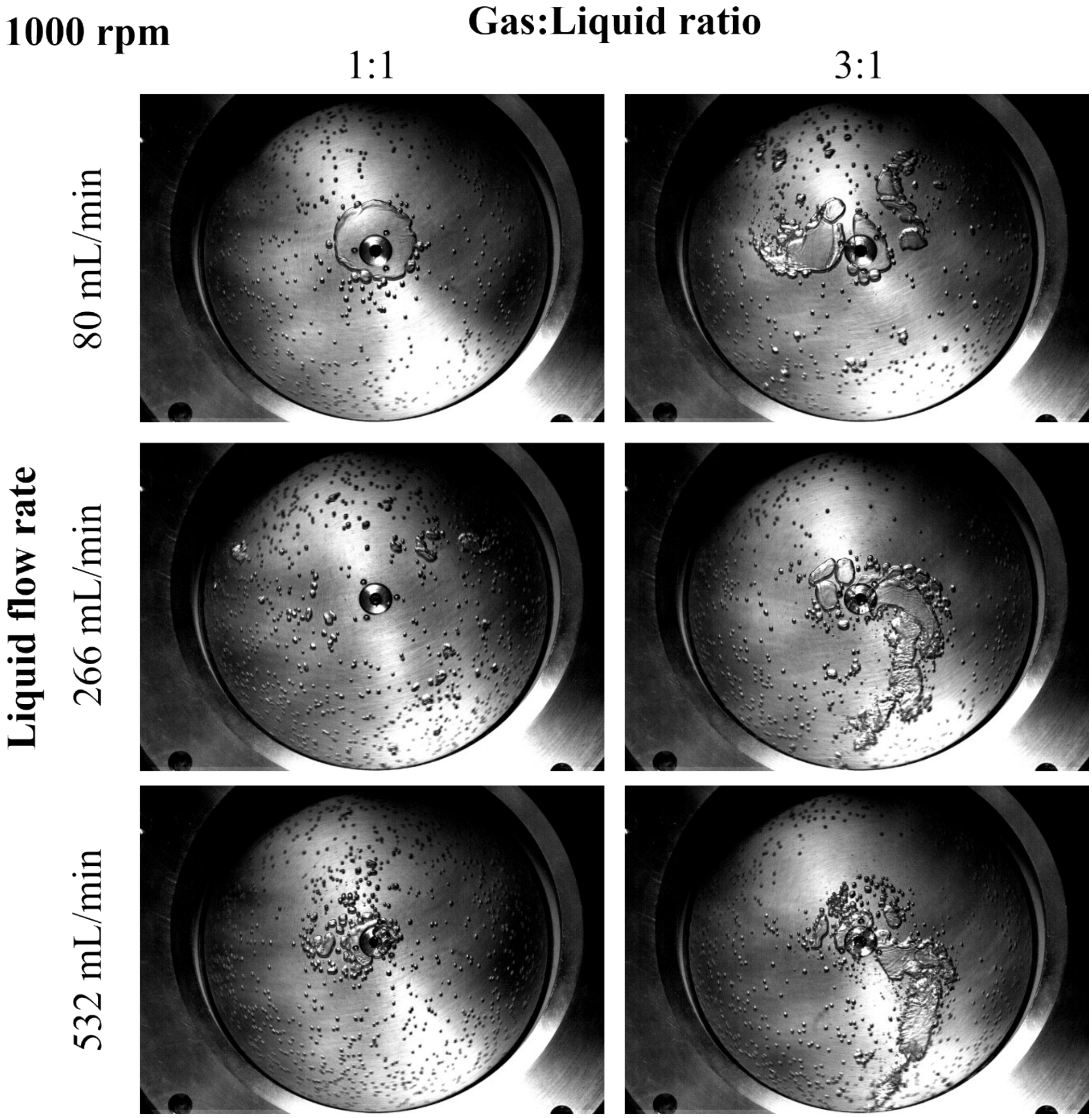

Figure S16 Representative images of the gas-liquid behavior under various flow rates and gas-liquid ratios, at a rotation speed of 1000 rpm.

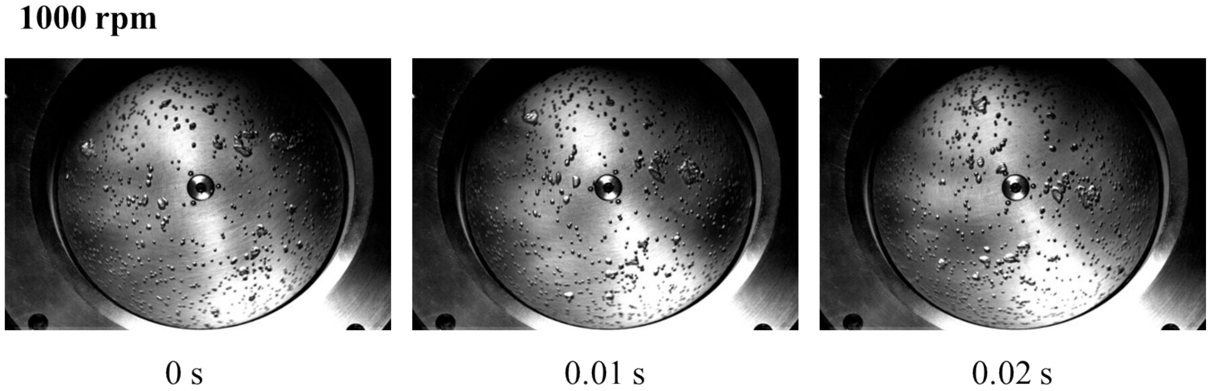

Figure S17 Timelapse of the irradiated part of the pRS-SDR at a rotation speed of 1000 rpm, liquid flow rate of 266 mL/min and 1 vol. equivalent of nitrogen, showing 0.33 rotation of the disk.

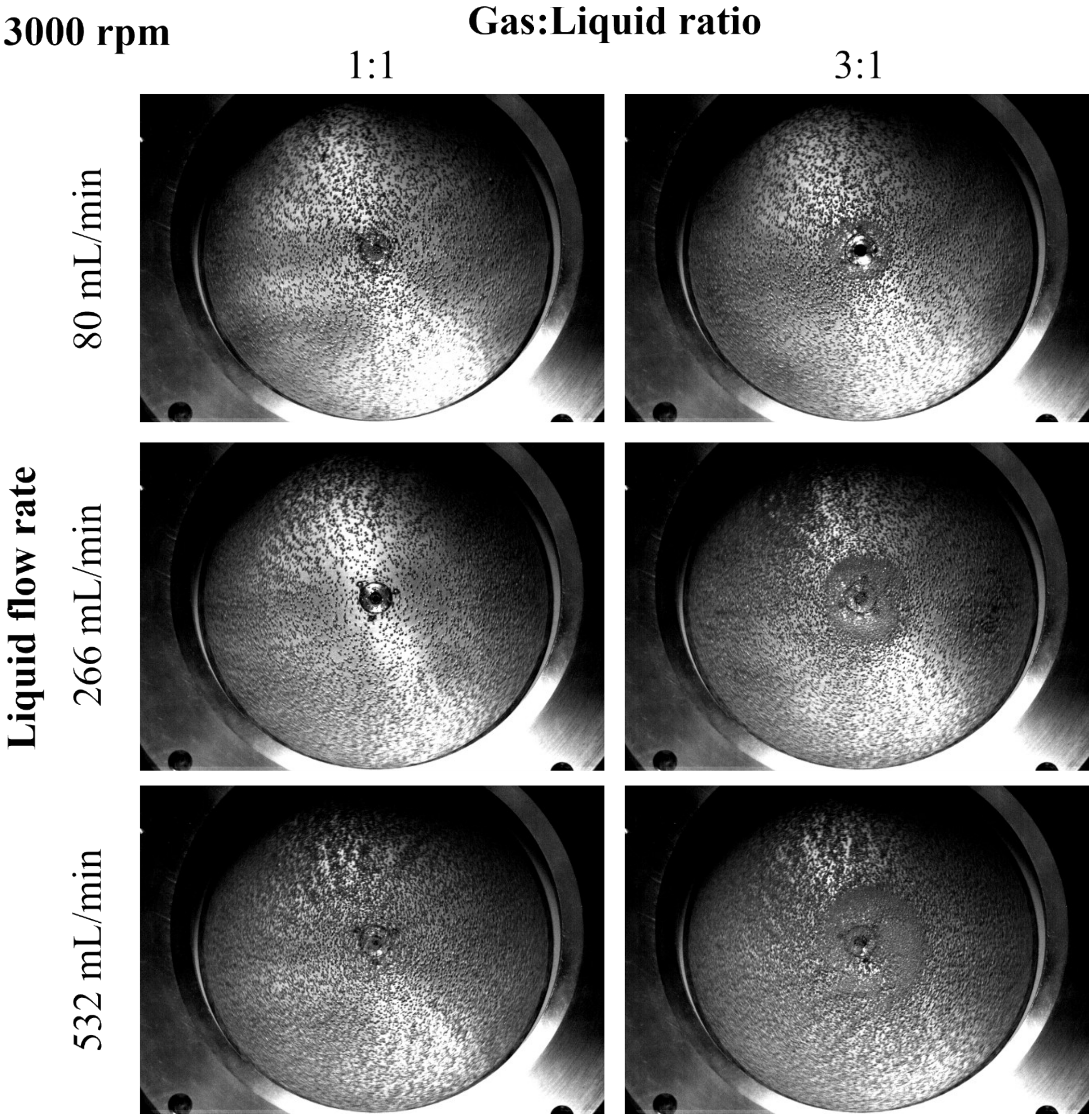

Figure S18 Representative images of the gas-liquid behavior under various flow rates and gas-liquid ratios, at a rotation speed of 3000 rpm.

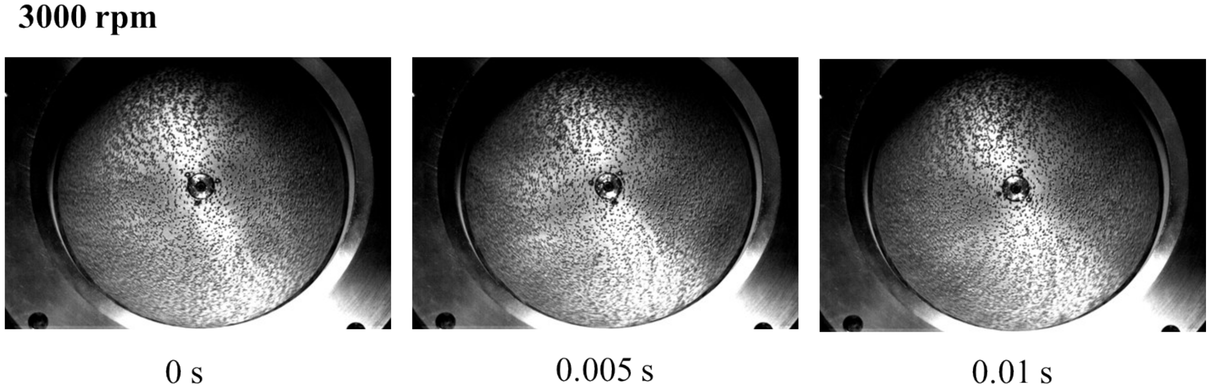

Figure S19 Timelapse of the irradiated part of the pRS-SDR at a rotation speed of 3000 rpm, liquid flow rate of 266 mL/min and 1 vol. equivalent of nitrogen, showing 0.50 rotation of the disk.

S5. Additional information pRS-SDR

S5.1 Reactor setup

The setup used to perform the actinometric experiments in the pRS-SDR is illustrated in Figure S20. The UV flood light has been extensively characterized in previous research.[1] The actinometric solution is supplied with a gear pump (VG 1000 digit) and the nitrogen flow is controlled with a MFC (Bronkhorst). The desired rotation speed can be set on the controller.

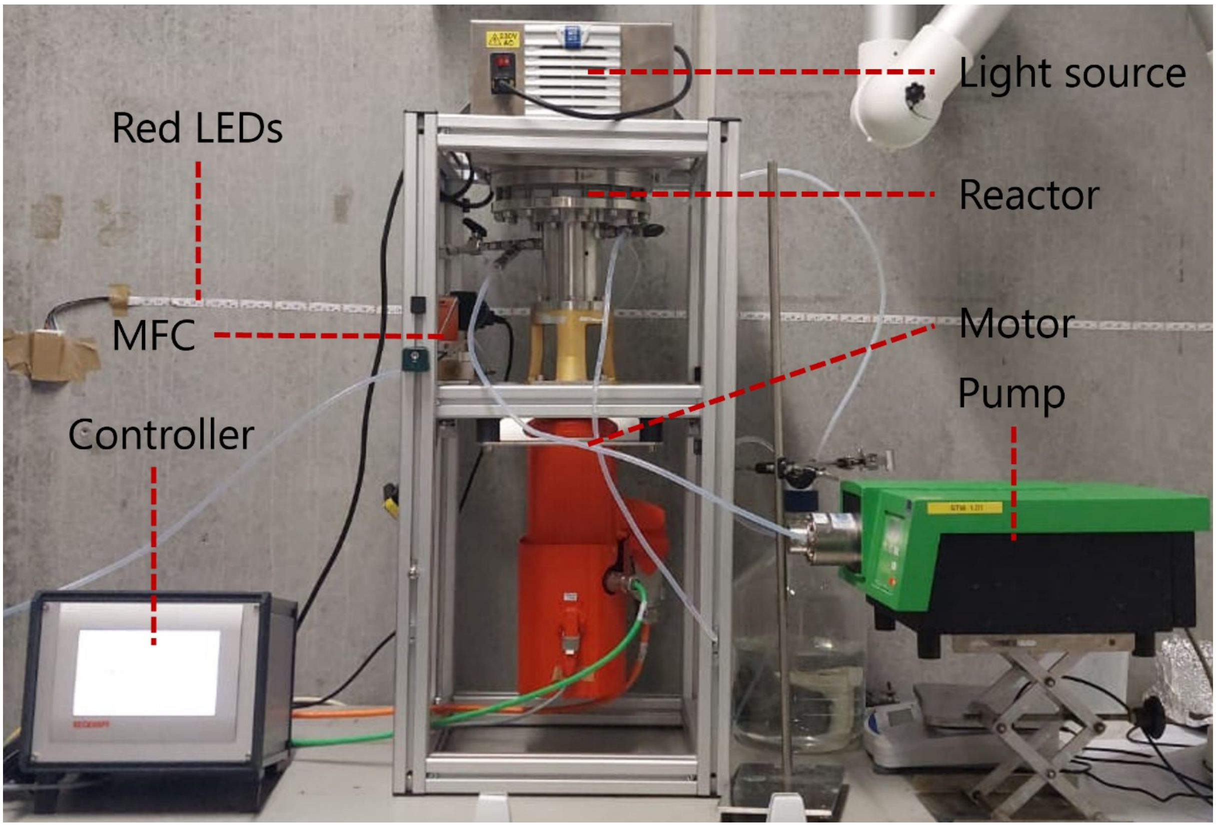

Figure S20 Overview of the setup used to perform actinometry experiments in the pRS-SDR reactor.

S5.2 Gas holdups

The gas holdup was measured using a gravimetric method.[17,18] Instead of the full reactor, a vessel with actinometric solution was positioned on a weighing scale. The vessel was used to fill the full reactor with liquid, whereafter the weight was noted. The liquid outflow was directed to the same vessel to allow for recirculation of the actinometric solution. When the desired rotation speed was reached, the gas and liquid flow rates could be set and the difference in weight was used to calculate the holdup over the full reactor, depicted in Figure S21. The overall gas holdup is relatively independent of liquid flow rate, but a dependency on rotation speed can be distinguished. Moreover, an effect of the volumetric equivalents of gas supplied to the system on the gas holdup can be noticed. The gas holdup for the full reactor represents the average of the irradiated part and non-irradiated part.

Large gas bubbles and low rotation speeds allow for the use of detailed image analysis to obtain the holdup in the irradiated part of the reactor. The workflow to obtain this information is shown in Figure S22. First, the relevant reactor volume is extracted from the frame. Hereafter, the volumetric amount of gas in this frame is determined. A boundary condition was set on the hue value of each pixel to distinguish between the liquid and gas phase. Assuming that the bubble spans nearly the full axial length of the reactor, allows for straightforward calculation of the gas holdup. This method was implemented in MATLAB 2022b and employed to videos (30 fps, made with a mobile phone camera) recorded over several residence times to obtain an average holdup, given in Figure S21b. Clearly, the gas holdup increases when changing the volumetric equivalents of nitrogen from one to three equivalents, indicating that on average larger or more bubbles are present in the irradiated part of the reactor.

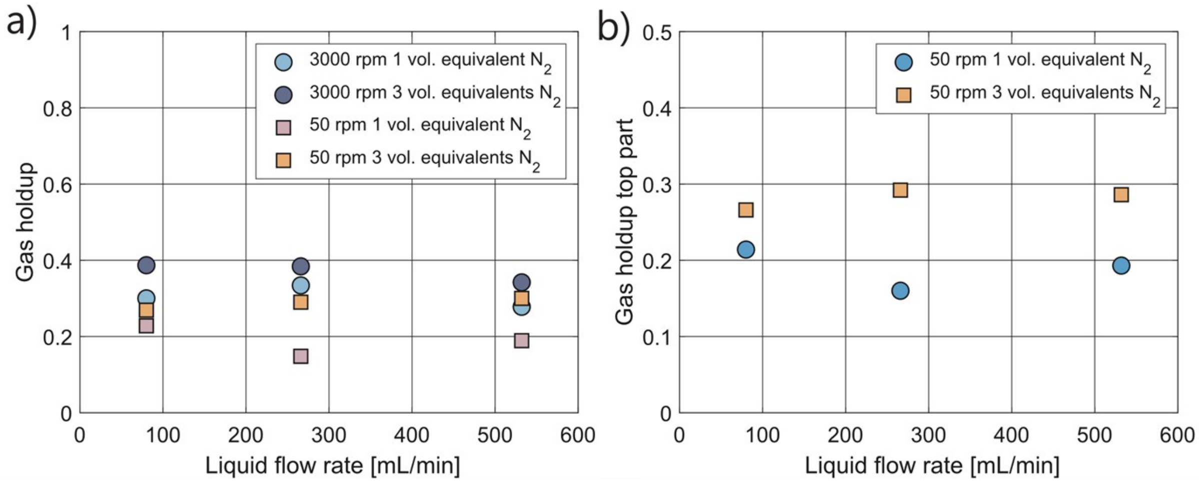

Figure S21 a) The average gas holdup of the pRS-SDR for the investigated rotation speeds at various gas and liquid flow rates. b) The gas holdup in the top part of the pRS-SDR for various conditions, determined by image analysis.

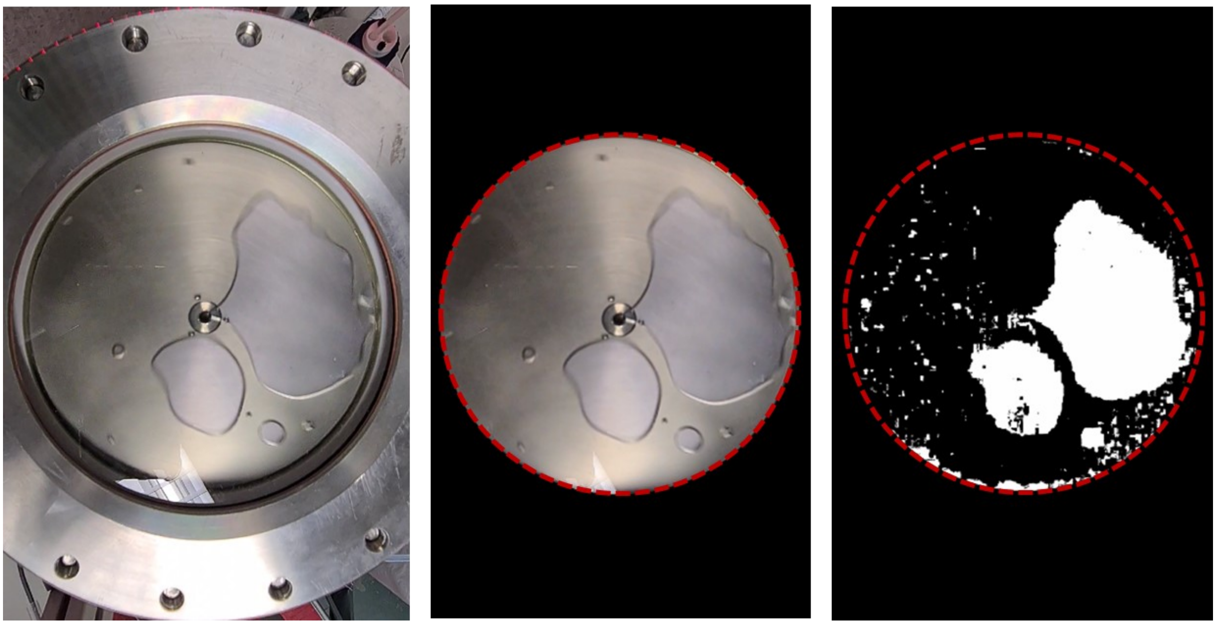

Figure S22 On the left, a representative image of the gas-liquid behavior at a rotation speed of 50, rpm. In the middle, the selected relevant volume, and on the right, the processed image indicating gas (white) and liquid (black).

Symbol list

Symbol	Description	Unit
C_A	Actinometer concentration	mol m^{-3}
$g_{p,\lambda}$	Spectral distribution function	-
$\bar{l}$	Effective optical path length	m
N_A	Amount of moles of actinometer	mol
N	Total number of CSTRs in series	-
$q_{n,p}$	Received photon flux	mol s^{-1}
t	Time	s
Greek symbols		
κ_A	Napierian absorption coefficient of actinometer	m^2 mol^{-1}
λ	Wavelength	m
τ	Residence time	s
φ	Quantum yield	-
Q_l	Liquid flow rate	m^3 s^{-1}
Subscript		
0	Initial	
i	Actual CSTR number	
l	Liquid	
p	Photon units	
n,p	Chemical amount basis	
λ	At wavelength λ	

References

1. Zondag, S. D. A. *et al.* Determining photon flux and effective optical path length in intensified flow photoreactors. *Nat. Chem. Eng.* **1**, 462–471 (2024).

2. Rabani, J., Mamane, H., Pousty, D. & Bolton, J. R. Practical Chemical Actinometry-A Review. *Photochem. Photobiol.* **97**, 873–902 (2021).

3. Hatchard, C. G. & Parker, C. A. A new sensitive chemical actinometer - II. Potassium ferrioxalate as a standard chemical actinometer. *Proc. R. Soc. London. Ser. A. Math. Phys. Sci.* **235**, 518–536 (1956).

4. Schuurmans, J. H. A. *et al.* Light-assisted carbon dioxide reduction in an automated photoreactor system coupled to carbonylation chemistry. *ChemRxiv* (2024). doi:https://doi.org/10.26434/chemrxiv-2024-sz6ng

5. de Beer, M. M., Keurentjes, J. T. F., Schouten, J. C. & van der Schaaf, J. Engineering model for single-phase flow in a multi-stage rotor–stator spinning disc reactor. *Chem. Eng. J.* **242**, 53–61 (2014).

6. de Beer, M. M., Pezzi Martins Loane, L., Keurentjes, J. T. F., Schouten, J. C. & van der Schaaf, J. Single phase fluid-stator heat transfer in a rotor–stator spinning disc reactor. *Chem. Eng. Sci.* **119**, 88–98 (2014).

7. Hop, C. J. W. *et al.* Hydrodynamics of a rotor–stator spinning disk reactor: Investigations by large-eddy simulation. *Phys. Fluids* **35**, 035105 (2023).

8. Martin, A. D. Interpretation of residence time distribution data. *Chem. Eng. Sci.* **55**, 5907–5917 (2000).

9. Pratley, C. *et al.* Development of a Horizontal Dynamically Mixed Flow Reactor for Laboratory Scale-Up of Photochemical Wohl–Ziegler Bromination. *Org. Process Res. Dev.* **28**, 1725–1733 (2024).

10. Maafi, M. & Brown, R. G. The kinetic model for AB(1ϕ) systems. *J. Photochem. Photobiol. A Chem.* **187**, 319–324 (2007).

11. Loubière, K., Oelgemöller, M., Aillet, T., Dechy-Cabaret, O. & Prat, L. Continuous-flow photochemistry: A need for chemical engineering. *Chem. Eng. Process. Process Intensif.* **104**, 120–132 (2016).

12. Wriedt, B. & Ziegenbalg, D. Application Limits of the Ferrioxalate Actinometer. *ChemPhotoChem* **5**, 947–956 (2021).

13. Masson, T. M., Zondag, S. D. A., Schuurmans, J. H. A. & Noël, T. Open-source 3D printed reactors for reproducible batch and continuous-flow photon-induced chemistry: design and characterization. *React. Chem. Eng.* **9**, 2218–2225 (2024).

14. Sender, M., Ziegenbalg, D., Wriedt, B. & Ziegenbalg, D. Radiometric measurement techniques for in-depth characterization of photoreactors – part 1: 2 dimensional radiometry. *React. Chem. Eng.* **6**, 1601–1613 (2021).

15. Cismesia, M. A. & Yoon, T. P. Characterizing chain processes in visible light photoredox catalysis. *Chem. Sci.* **6**, 5426–5434 (2015).

16. Demas, J. N., Bowman, W. D., Zalewski, E. F. & Velapoldi, R. A. Determination of the quantum yield of the ferrioxalate actinometer with electrically calibrated radiometers. *J. Phys. Chem.* **85**, 2766–2771 (1981).

17. Chaudhuri, A. *et al.* Process intensification of a photochemical oxidation reaction using a Rotor-Stator Spinning Disk Reactor: A strategy for scale up. *Chem. Eng. J.* **400**, 125875 (2020).

18. Manzano Martínez, A. N., Chaudhuri, A., Besten, M., Assirelli, M. & van der Schaaf, J.

Micromixing Efficiency in the Presence of an Inert Gas in a Rotor–Stator Spinning Disk Reactor. *Ind. Eng. Chem. Res.* **60**, 8677–8686 (2021).
